# Supplementary material for: Memristive tonotopic mapping with volatile resistive switching memory devices
Source: Nat Commun. 2024 Apr 1;15:2812. doi: 10.1038/s41467-024-47228-1 (PMC10985068; doi:10.1038/s41467-024-47228-1)
Supplement: Supplementary file 1 — Supplementary Information [file 41467_2024_47228_MOESM1_ESM.pdf]

Supplementary material for the manuscript:

"Memristive tonotopic mapping with volatile resistive switching memory devices"

Alessandro Milozzi, Saverio Ricci & Daniele Ielmini

Dipartimento di Elettronica, Informazione e Bioingegneria, Politecnico di Milano,

Piazza Leonardo da Vinci 32, 20133, Milan, Italy.

E-mail: daniele.ielmini@polimi.it

Supplementary note 1: Experimental setup and measurement procedure

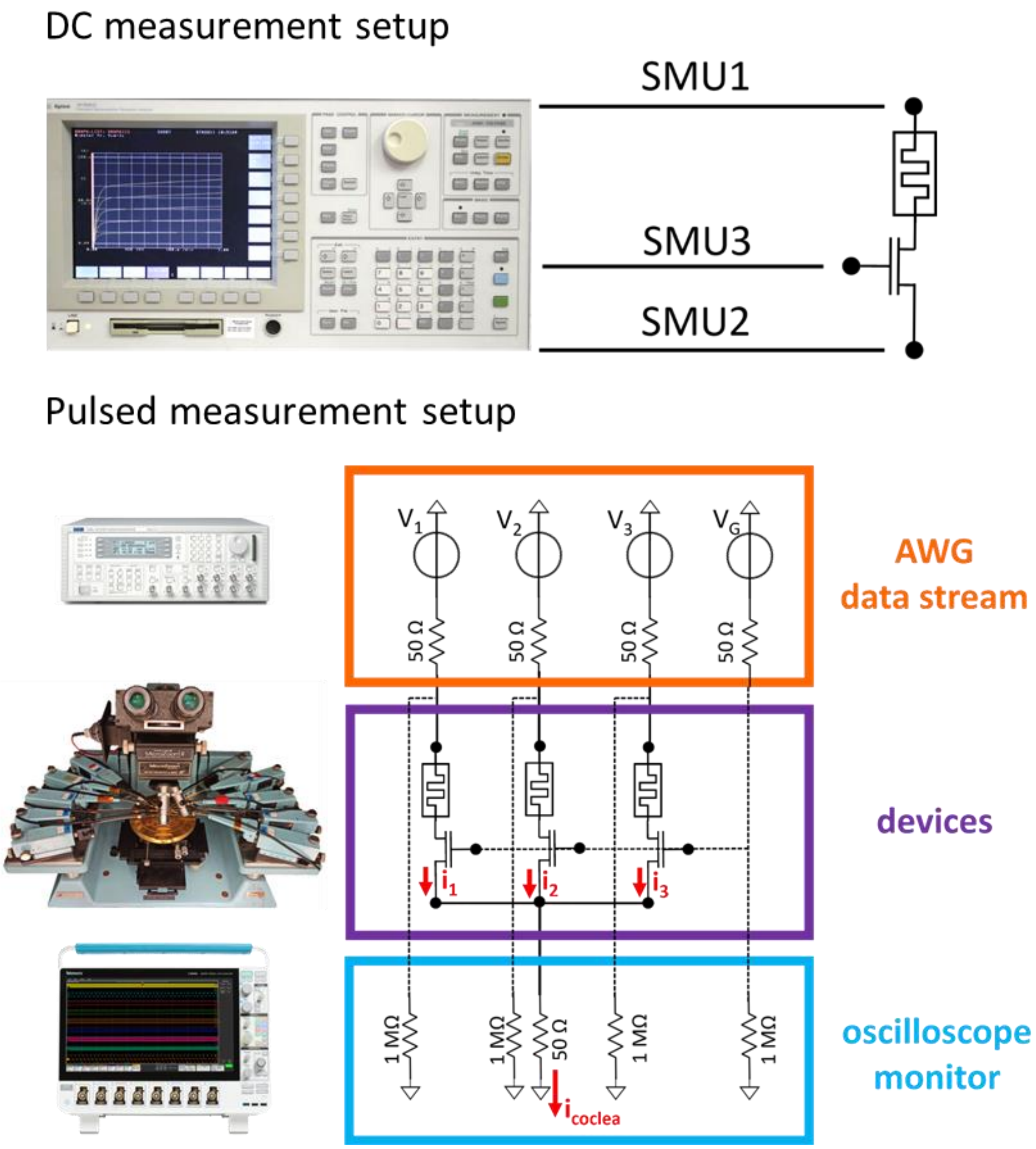

Supplementary Figure 1: Measurement setup. Devices are contacted with tungsten needles using probe station inserted in a Faraday cage connected with external instrumentation through coaxial cables. An arbitrary waveform generator (AWG) is used for pulsed measurements, and AWG channels are set to have R=50 Ω termination. The oscilloscope is used to monitor the temporal traces and the channels have R=50 Ω for the current measurement and R=1 MΩ for the voltage monitoring. All the applied voltages are monitored from AWG during the experiment to have the stream history. Gate is kept constant during the experiment with 1 V amplitude.

Supplementary note 2: MOSFET characteristics

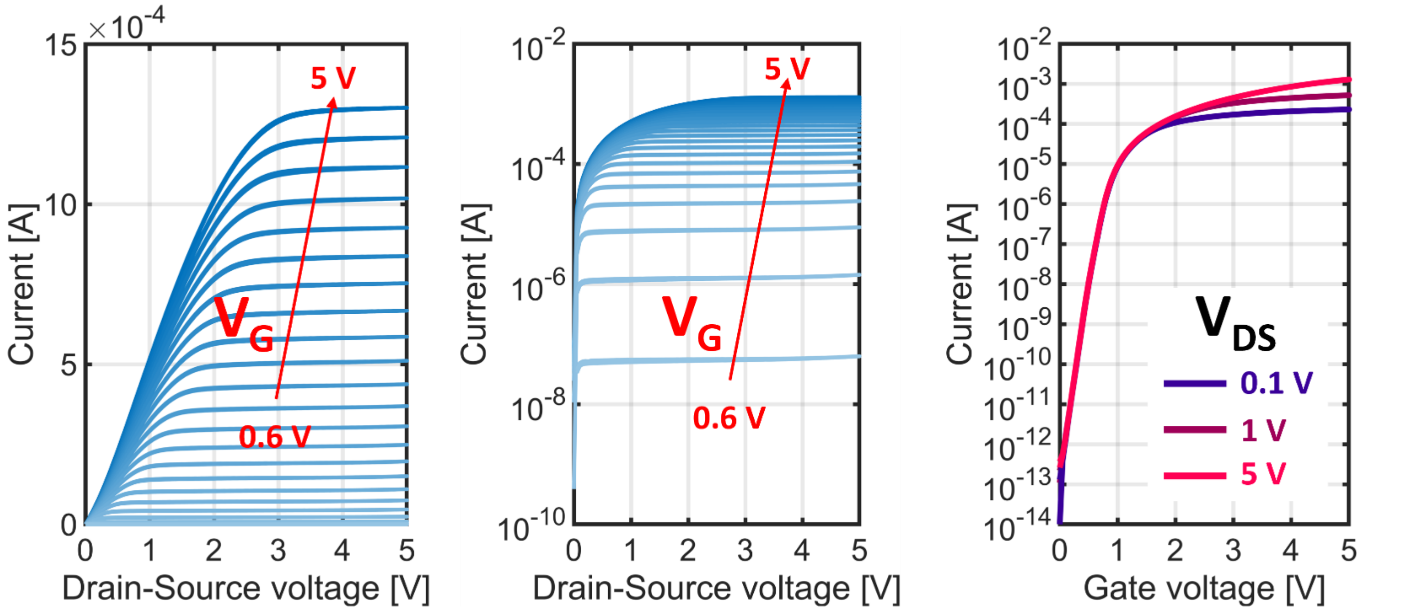

Supplementary Figure 2: MOSFET characterization. The transistor adopted in this work is a third-party device based on standard CMOS fabrication process. Here is reported the transfer characterization (in linear and logarithmic scale) and the transcharacteristics for different V_{DS}.

Supplementary note 3: DC and pulsed electrical characterization of device-to-device and cycle-to-cycle variability

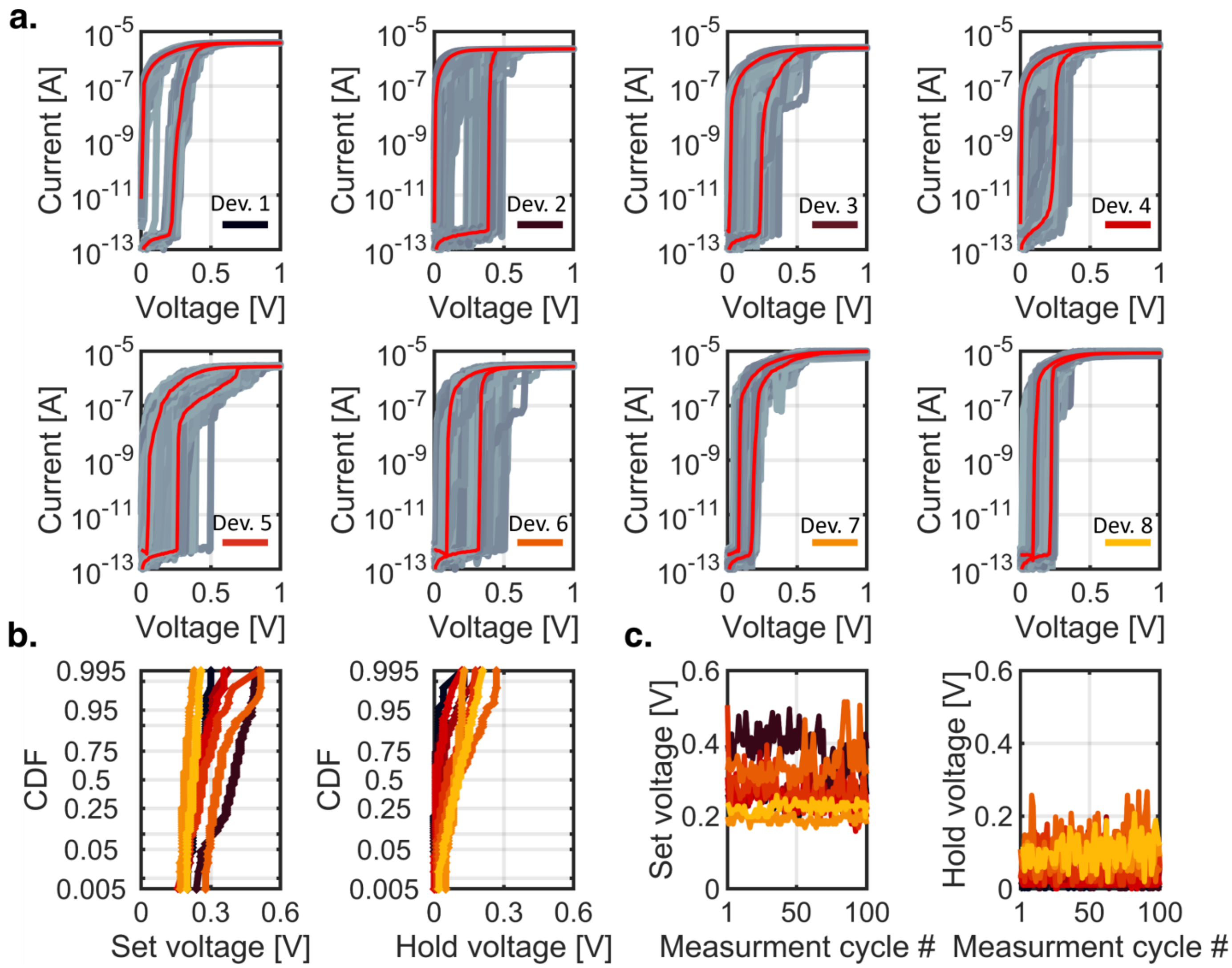

Supplementary Figure 3: DC characterization of device-to-device and cycle-to-cycle variability. a. I-V curves for 8 devices in quasi-static regime with 100 cycles carried out with Agilent 4156C semiconductor parameter analyzer, with a compliance current between 1 μA and 10 μA to protect the devices from failure and possible permanent short circuit. In red, an example of a representative I-V curve of the specific device **b.** Distributions of set voltage and hold voltage: the device-to-device variability (D2D) is not significant for our purpose, with set voltages always between 0.2 and 0.4 with different distributions. During the characterization devices with larger parameters were not found. The hold voltage (considered as the voltage at which the current is less than 10 nA) has a similar behavior. The cycle-to-cycle variability (C2C) shows broadened distributions for each specific device and the presence of a large variance is responsible for the switching probability properties used as a backbone of the presented work. **c.** The evolution during the cycling of the I-V curves is presented, considering that an I-V curve has an overall duration of 3-5 seconds. Both the set and the hold voltages are stable within a range of values, without degradation toward higher or lower values.

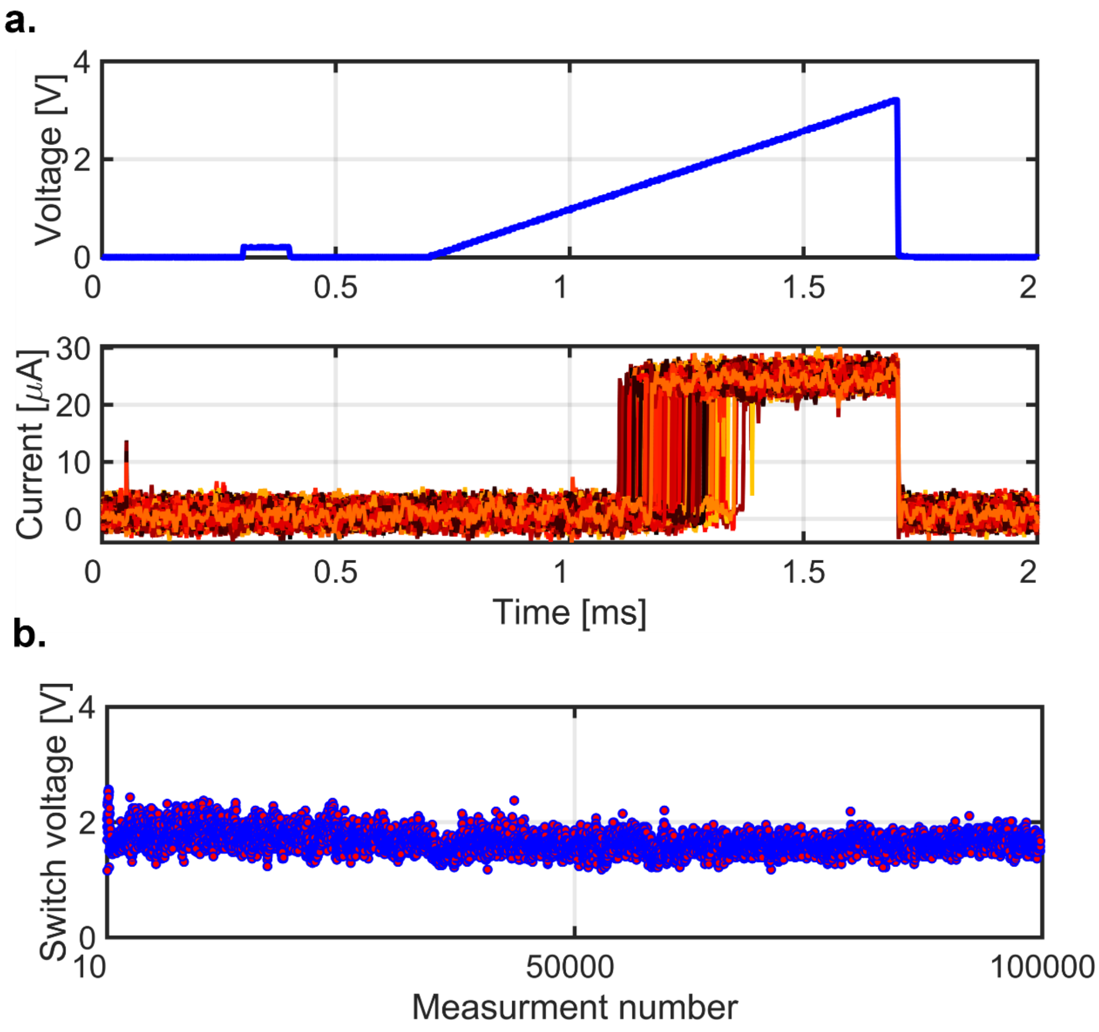

Supplementary Figure 4: Pulsed characterization with triangular pulses. a. Voltage pulse and 100 current traces as a result of the stimulation. A 200 mV initial reading pulse is applied to check if the device is broken, and thus stuck in LRS, or correctly in HRS. **b.** The behavior of the set voltage versus the measurement number. 100k cycles are applied to monitor the degradation, showing no significant deviation in the value. Since the pulse has a triangular shape (used to retrieve the voltage at which the switch happens), the applied electric field is gradual with respect to the case of the square pulse (which is used in the experiments), and thus the value of the switching voltage is higher with respect to the distributions proposed in the text.

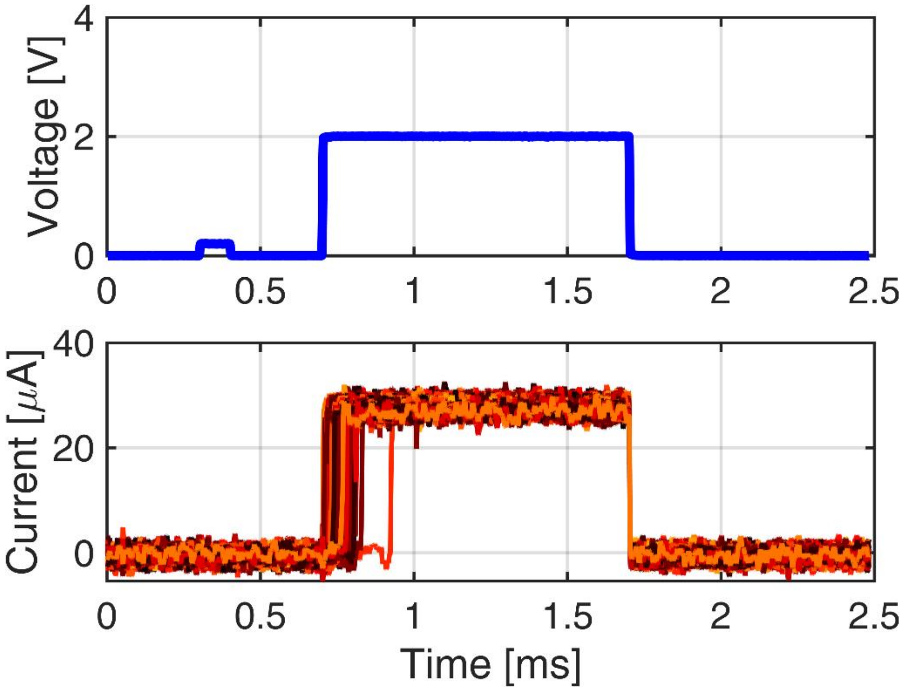

Supplementary Figure 5: Rectangular voltage pulse and relative current response for 100 cycles. The voltage value cannot be retrieved in this case due to a step-like jump from 0 V to 2 V.

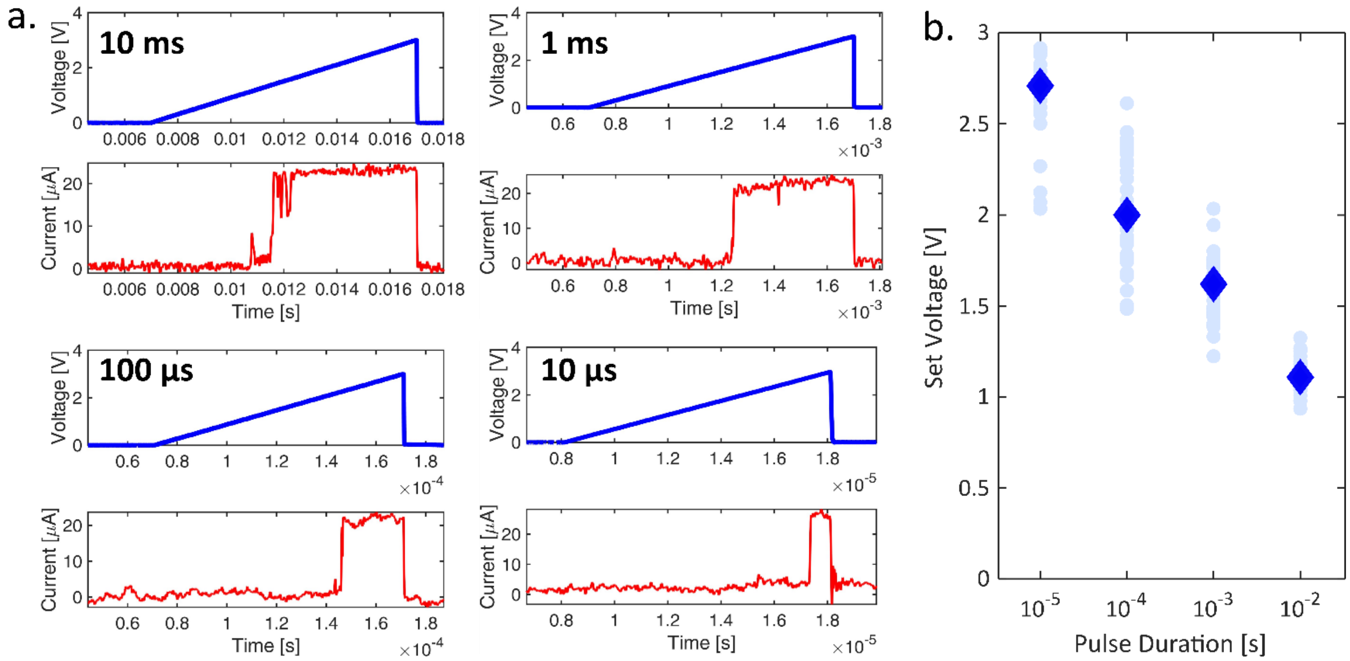

Supplementary Figure 6: Impact of pulse duration on set voltage. a. Triangular voltage pulses with different time duration and associated current response. **b.** Set voltage versus pulse duration: set voltage decreases increasing exponentially the time duration of programming pulse (accordingly to the so-called "Voltage-time dilemma")

Supplementary note 4: Threshold voltage variability in Ag-based volatile memristors

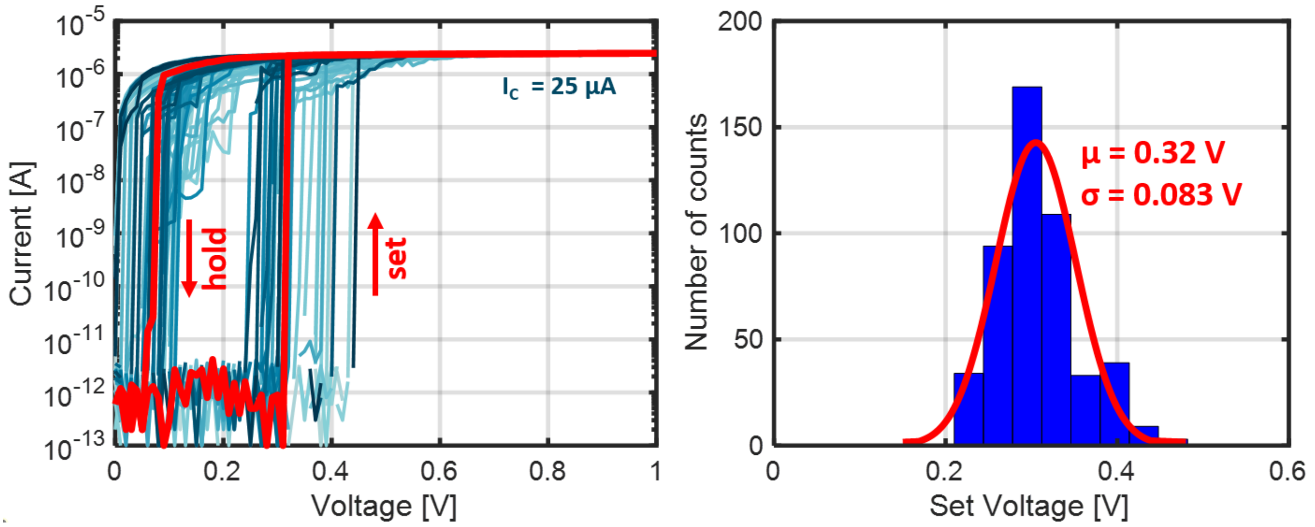

Supplementary Figure 7: Threshold voltage variability. The images report the I-V curve performed in quasi static measurements and the distribution of the set voltage. On the left 20 cycles as example of the electrical response. The devices show small cycle-to-cycle variability, with repeatable behavior. On the right the distribution of the set voltage and a Gaussian fitting with distribution parameters.

Supplementary note 5: Relaxation time

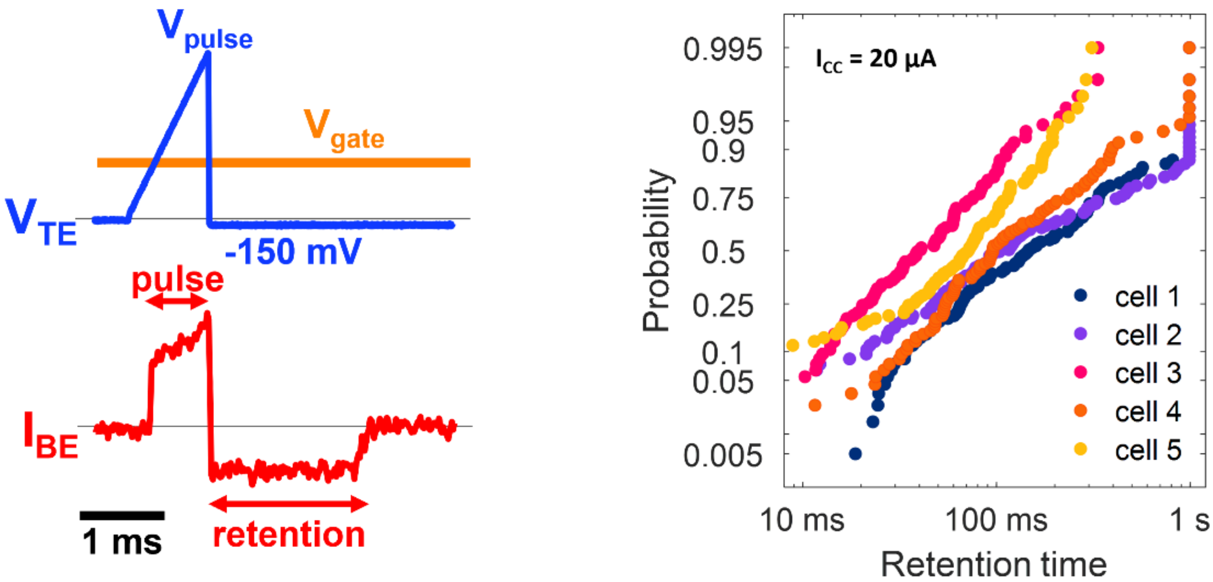

Supplementary Figure 8: Time characterization of the retention of the filament. On the right, after a 5 V amplitude triangular pulse to switch on the cell with an I_C = 20 µA (through the saturation regime of the transistor, tuning V_g), a constant reading voltage of -150 mV is applied to monitor the retention. On the left, comparison between the retention distributions of the 5 memristor, with an I_C = 20 µA. The distributions are clamped to 1 s due to the measurement conditions. 1 ms pulse with 3 V amplitude is applied, followed by -100 mV constant reading.

Supplementary note 6: Voltage pulse waveforms and current responses for different voltage amplitudes

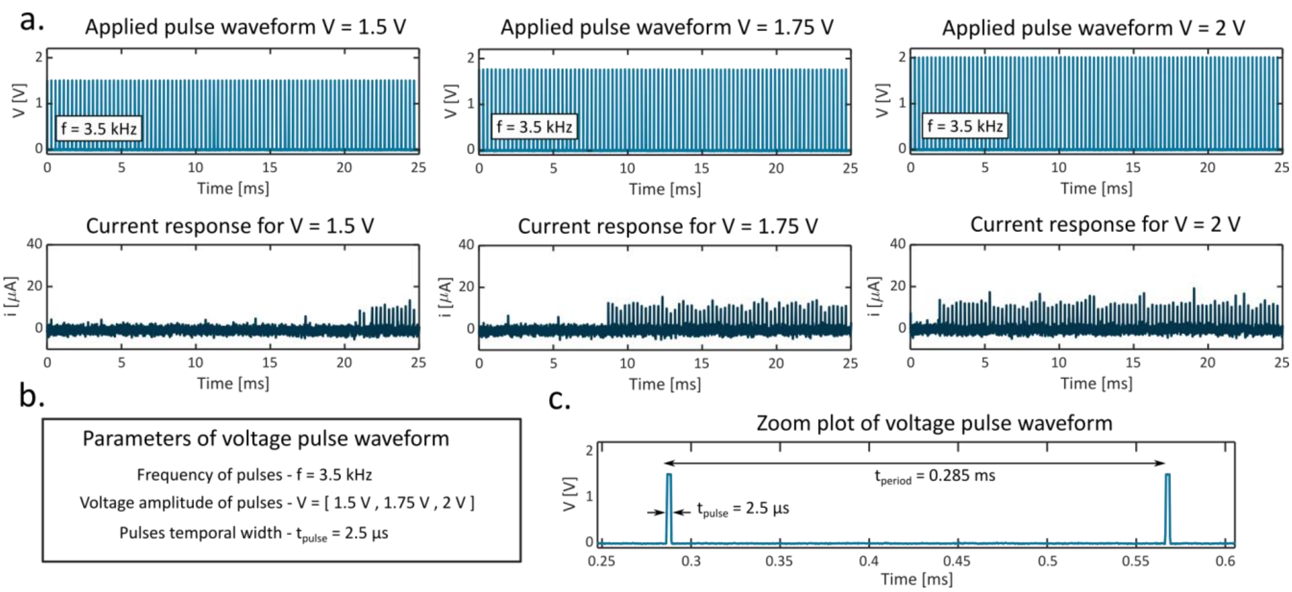

Supplementary Figure 9: Current responses to trains with different voltage amplitudes. a. Applied voltage pulse waveforms and relative current responses for different voltage amplitudes. **b.** Parameters of the voltage pulse waveform. **c.** Zoom of the voltage pulse waveform.

Supplementary note 7: Measurement procedure for the characterization of switching probability

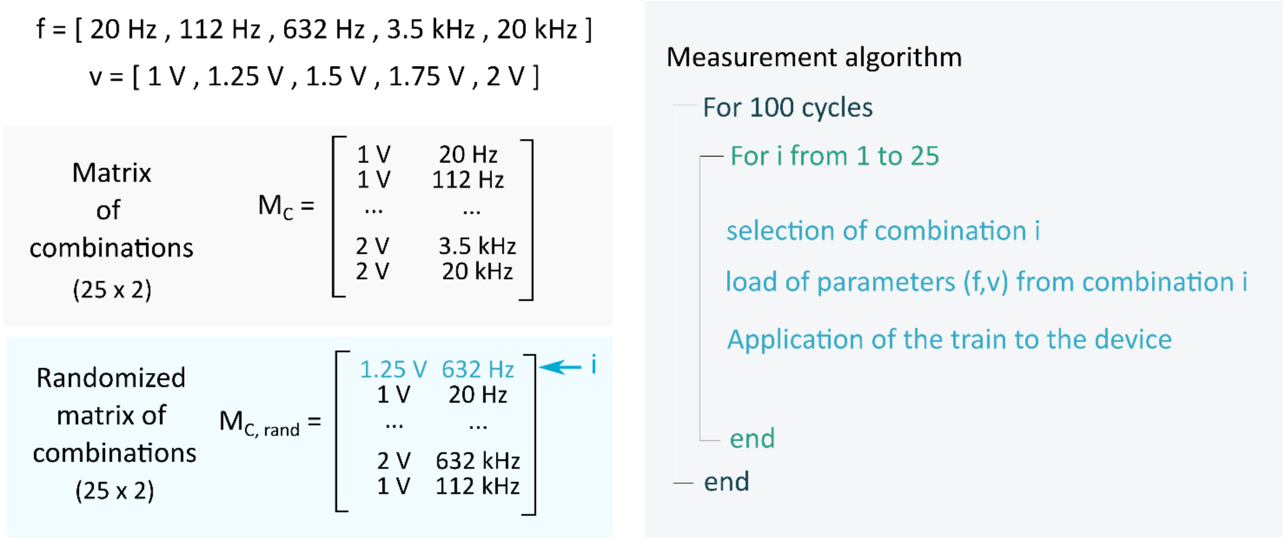

Supplementary Figure 10: Measurement protocol for switching probability characterization to avoid correlation effects.

Supplementary note 8: Switching probability variability

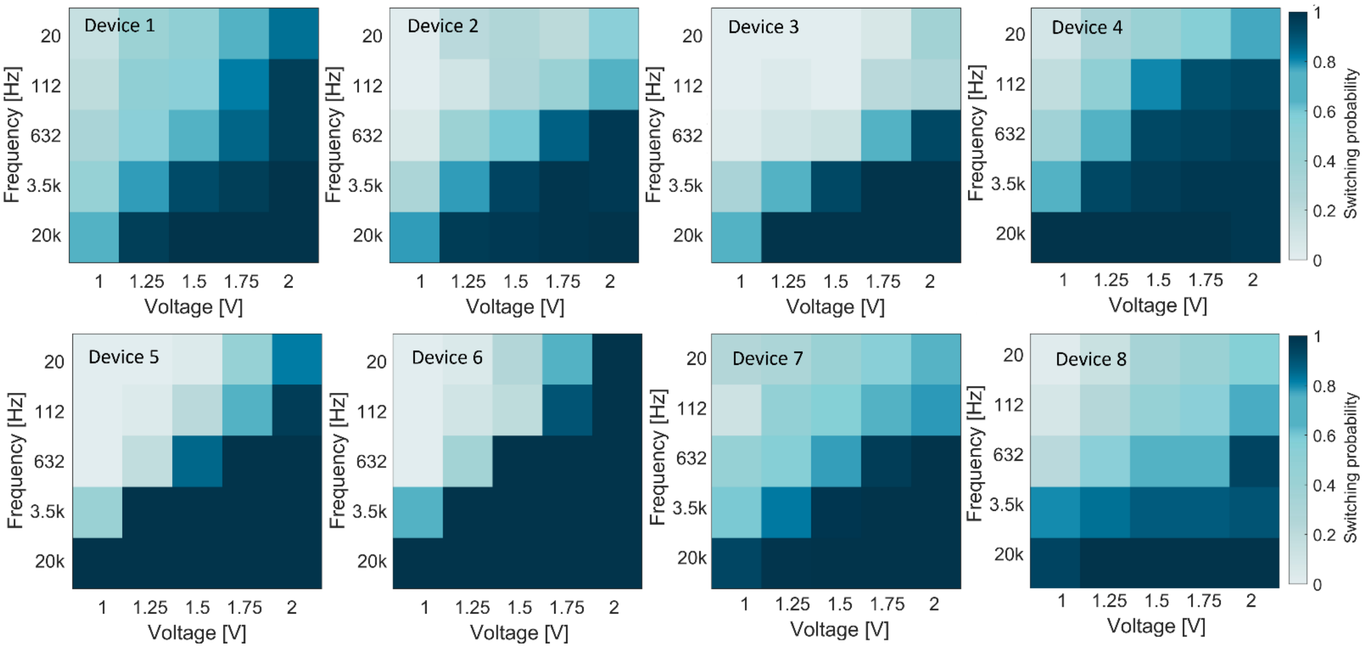

Supplementary Figure 11: Characterization of switching probability for 8 different devices

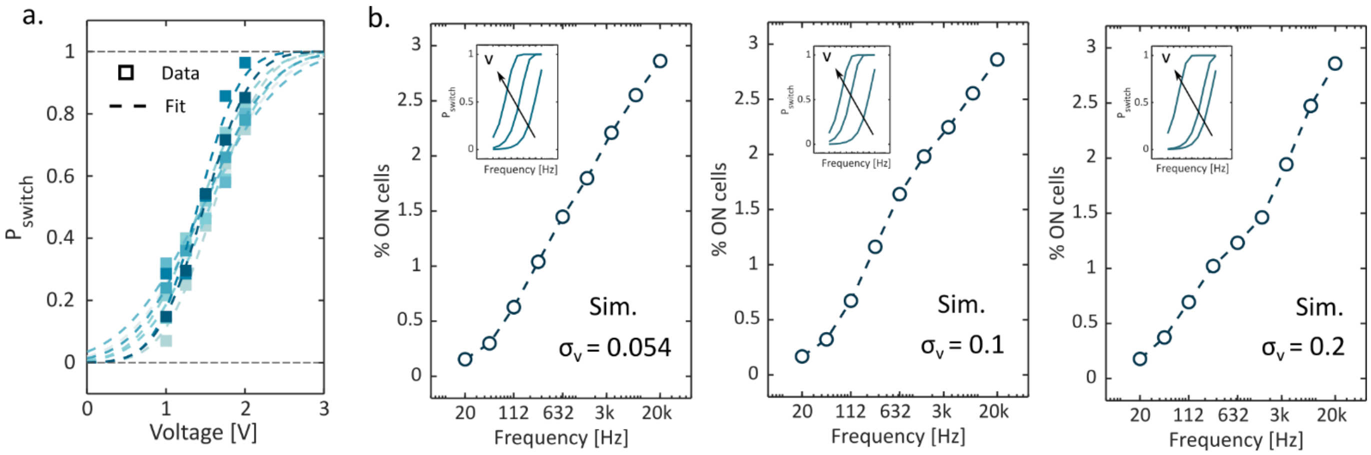

Supplementary Figure 12: Impact of variability on frequency sensing a. Switching probability curve for 10 different devices (f=632 Hz): there is a natural variability of the curve that can be captured through the characteristic voltage Θ = V(Pswitch=0.5) that has a standard deviation equal to σv = 0.054. **b.** Impact of the variability of switching probability on frequency sensing circuit of Fig.3: Simulation agrees with experimental results as shown in Supplementary Figure 16. If the variability increases, the linearity of the curve starts to be impacted, still well performing for frequency sensing on a logarithmic scale.

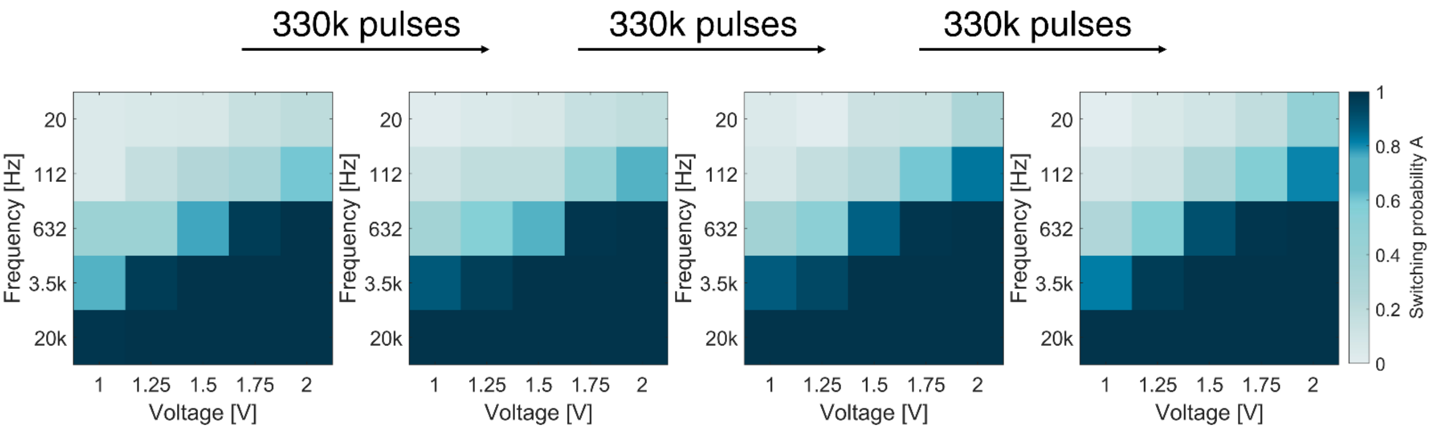

Supplementary Figure 13: Evolution in time of switching probability on the same device after cycling.

Supplementary note 9: Raw experimental current traces

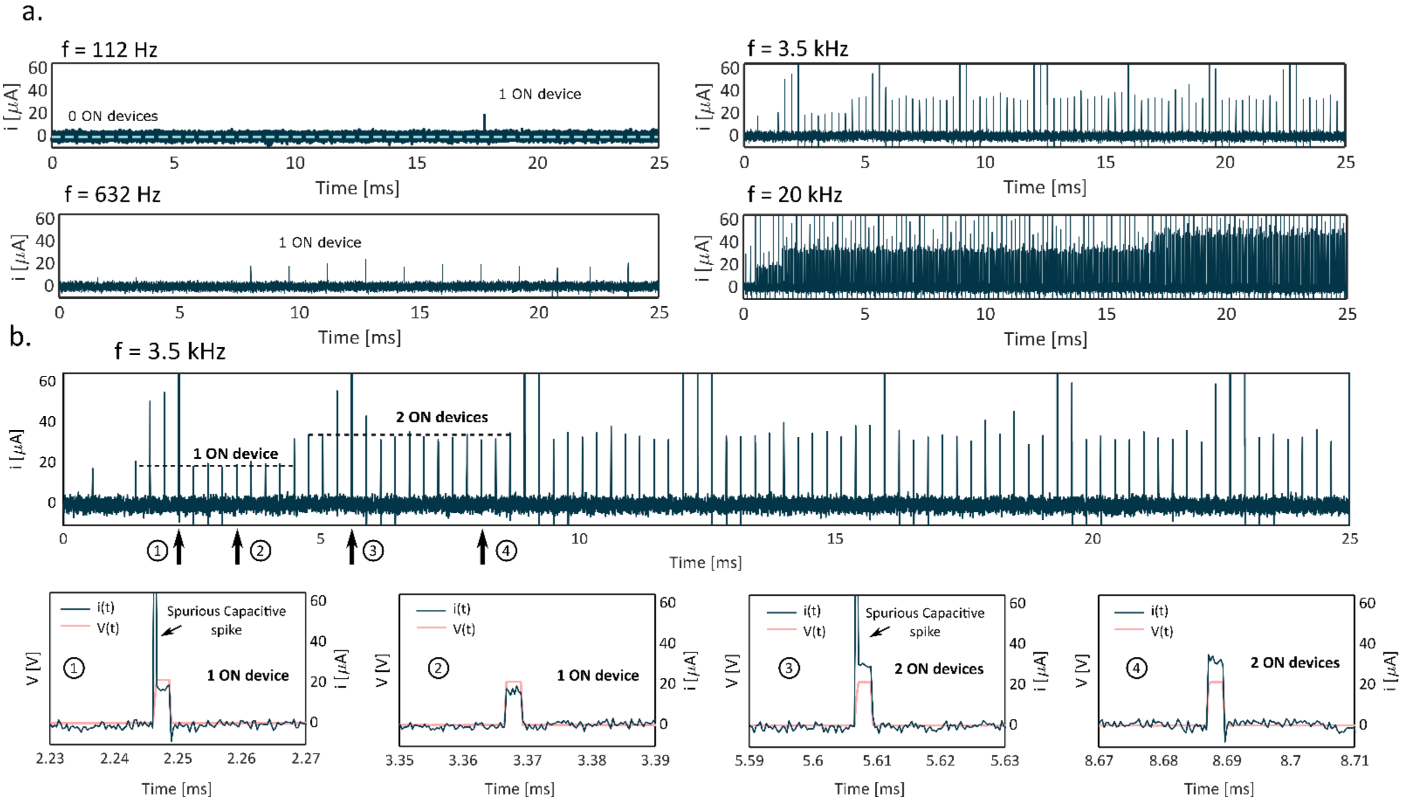

Supplementary Figure 14: Examples of raw current traces. a. Raw current traces corresponding to the smoothed traces of Fig. 3 show spurious capacitive spikes due to experimental setup (coaxial cables, instrumentation, chuck, manipulators). **b.** Four closer looks at individual spikes within the raw current trace with f=3.5 kHz, focusing on a 40 µs time window: spurious capacitive spikes involve just the first point of the programming pulse when present going toward a fast discharge compared to the pulse duration (2.5 µs), so they can be easily filtered through a low pass filter.

The averaged current traces of Fig.3b of the main text are associated with the raw current traces of Supplementary Figure 14a. Also in the raw traces is possible to see different current levels corresponding to a different number of ON devices but with the contamination of spurious capacitive spikes. In fact, being the pulse duration equal to 2.5 µs, it is difficult to recognize device switching from spurious capacitive spikes by watching the raw traces in the time scale of tens of milliseconds: they can be visually distinguished just thanks to a temporal scale zoom as shown in Supplementary Figure 14b.

These spurious spikes are due to a capacitive-coupled disturbance referable to the experimental setup (coaxial cables, manipulators, chuck, instrumentation, etc.). It is possible to readily eliminate them through a simple low pass filter (in our case a mobile median filter with 7 points time window) thereby enabling accurate extraction of the device's conductance as demonstrated in Fig. 2b, and of the device’s state as proved in the histograms of Fig. 3c. It is important to note that this disturbance effect would not be encountered in a fully integrated version of the chip, eliminating the coupling sources for the disturbance.

Supplementary note 10: Stochastic model for switching probability

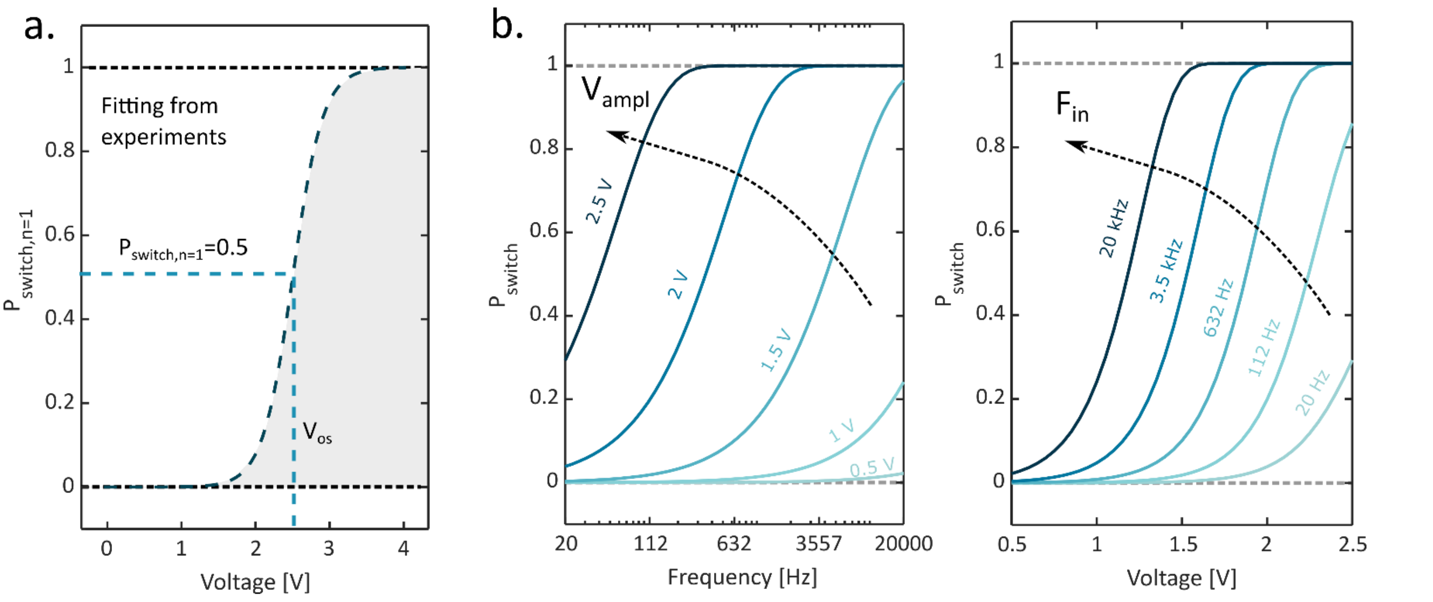

Supplementary Figure 15: Switching probability model a. Switching probability with single pulse as function of voltage amplitude. Experimental curve is fitted with Fermi function: offset voltage and β are the extracted parameters used in the stochastic model. **b.** Results of simulations with different operative conditions i.e. voltage amplitude and frequency.

Experimental results for switching probability are explained through pure probabilistic assumptions. The starting point for the model is the probability to siwtch on the device with a single pulse. As reported in Supplementary Figure 15b, this probability is function of the voltage amplitude of the programming pulse V_{in}. We can describe this curve with a Fermi function:

$$P_{switch}(V_{in})|_{n=1} = \frac{1}{1+e^{\frac{-(V_{in}-V_{os})}{\beta}}} \quad (1)$$

Where V_{os} and β are parameters extracted from the fitting of the experiment. We need to generalize the switching probability referring to a train of spike meaning that:

$$P_{switch} = P_{switch}(V_{in}, f_{in}, T_{window}) \quad (2)$$

Where f_{in} is the frequency of the voltage pulses and T_{window} is the time window of observation. We know the probability of success (to switch on the device) with a single trial (pulse) i.e., n=1 from Supplementary Equation (1). The probability of success with n trials is equal to:

$$P_{switch}(V_{in}, n) = 1 - P_{NO\ switch}(V_{in}, n) = 1 - (\ 1 - P_{switch}(V_{in})|_{n=1}\ )^{n} \quad (3)$$

Given a train with a specific frequency and time window, the number of pulses i.e., the number of trials is eq

$$n = f_{in} T_{window} \quad (4)$$

We can now express the relationship for Supplementary Equation (2) combining Supplementary Equations (3) and (4):

$$P_{switch}(V_{in}, f_{in}, T_{window}) = 1 - (\, 1 - P_{switch}(V_{in})|_{n=1}\,)^{f_{in}T_{window}} \quad (5)$$

The description of the generalized probability of switching through Supplementary Equation (5) is experimentally validated as reported in Supplementary Note 11, both for single device and parallel devices experiments ($n_{devices}$=3) of Fig 3. Device to device variability can be simply included through distributions for V_{os}.

Supplementary note 11: Stochastic model validation

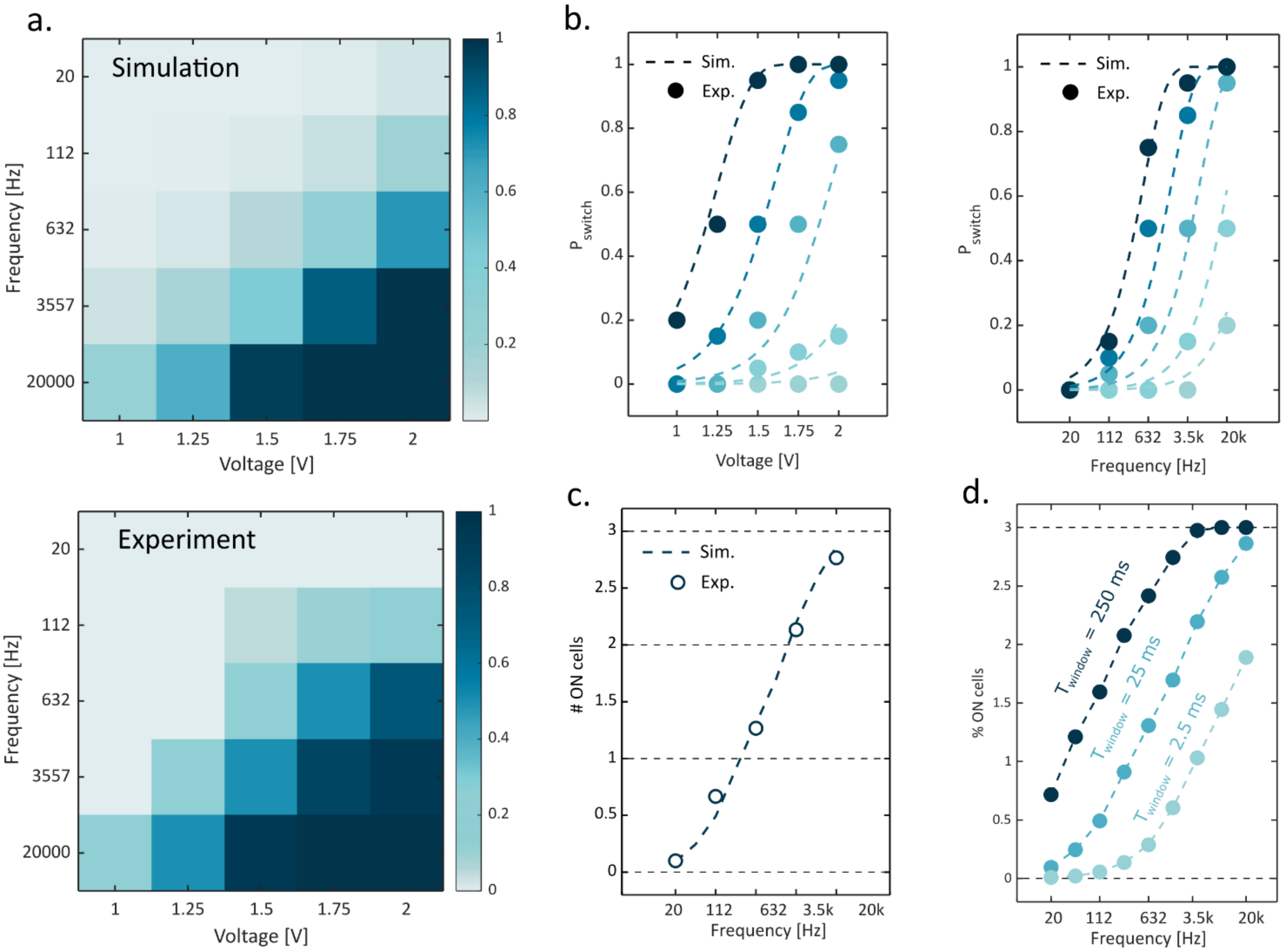

Supplementary Figure 16: Stochastic model validation under different operative conditions. a. Comparison between experiment and results of simulation from model reported in Supplementary note 10 for different operative conditions. **b.** Simulated P(v) and P(f) curves for different operative conditions compared to experimental data **c.** Comparison between simulation results and experiment of 3 parallel devices. **d.** Simulation results for 3 parallel devices with different time windows: the model enables to study the system with different operative conditions.

Supplementary note 12: ON/OFF region detection with XOR logic

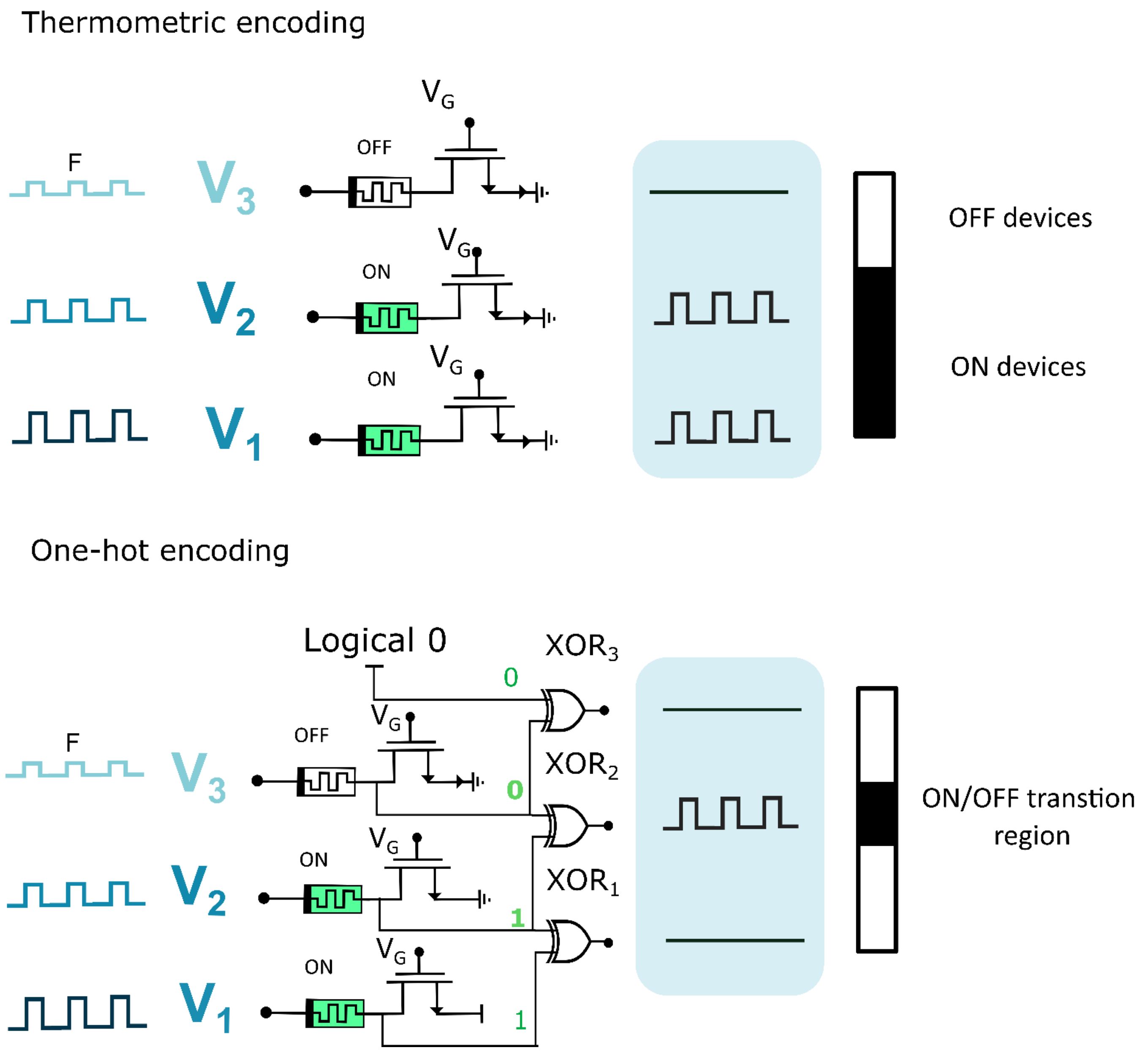

Supplementary Figure 17: Thermometric encoding of parallel volatile memristors system vs one-hot encoding with XOR logic.

The system of parallel volatile memristors can sense the frequency of the incoming trains as demonstrated in the main text. However, cells start to switch on from the bottom of the schematic i.e., from the devices with higher voltage amplitude. The most probable configuration is that we can divide the devices into a region of ON devices and OFF devices as depicted on the top schematic. This corresponds to a thermometric encoding of the frequency as in the top of the Supplementary Figure 17: the higher is the input frequency, the higher is the number of ON devices. This experimental result is reported in Figure 3 of main text. To attribute a single spatial location to a specific input frequency we can use a XOR logic to detect the transition region between the last ON device and first OFF device as reported in the bottom. Thanks to this system, we have that the normally-active XOR is just one referred to a specific spatial position i.e., one-hot encoding.

Supplementary note 13: Different number of elements for speech recognition network

Our choice of three XOR outputs was made considering that the number N_{OUT} of XOR outputs is linked to the number of memristive devices for each memristive tonotopic map (MTM) needed for the cochlear processing and so to the number of required channels in the analog-to-spike conversion. Moreover, N_{OUT} also determines the number of weights of the cortical network for interpretation being the number of weights of the first layer equal to $N_{W1} = N_{OUT} \times 64$ as illustrated in Supplementary Figure 18. We have kept N_{OUT} as low as possible to showcase the capabilities of our system with a minimal number of elements, thus gaining in power consumption and occupied area, while still ensuring excellent accuracy in classification of 96.5%. This value, in fact, is in the order of best automatic speech recognition systems (ASR) implemented with deep learning (DL) techniques at the edge [12]. Here, we have a limited set of words compared to the ones involved in the DL-ASR and a direct comparison is out of scope. However, the crucial point is to determine a threshold for the absolute value indicating good accuracy in ASR that is around 95%, showing that $N_{OUT} = 3$ is a good value to reach a proper accuracy. For the up-scaling of the system, a larger set of spoken words would certainly benefit from a greater N_{OUT}, becoming the increment essential to deal with a substantial increase in the dataset.

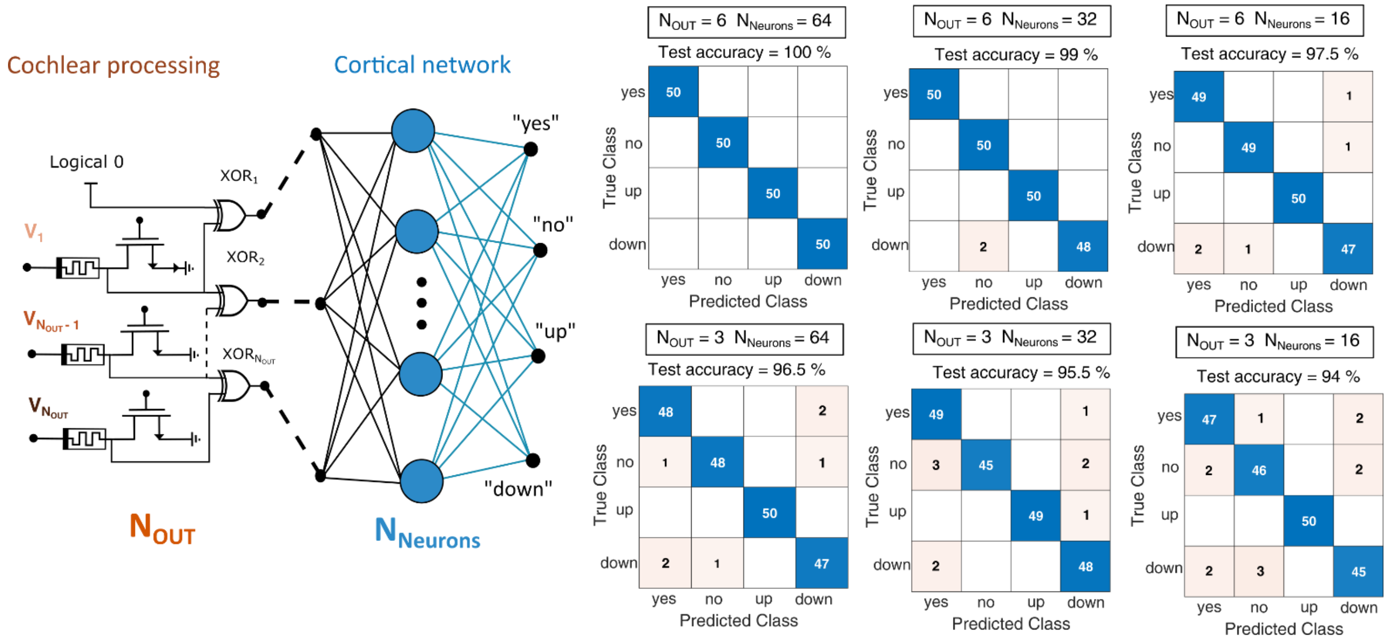

Supplementary Figure 18: Simulation of different configurations changing the number of XORs = N_{out} and the number of neurons of cortical network and the related impact on accuracy.

Supplementary note 14: analog-to-spike conversion

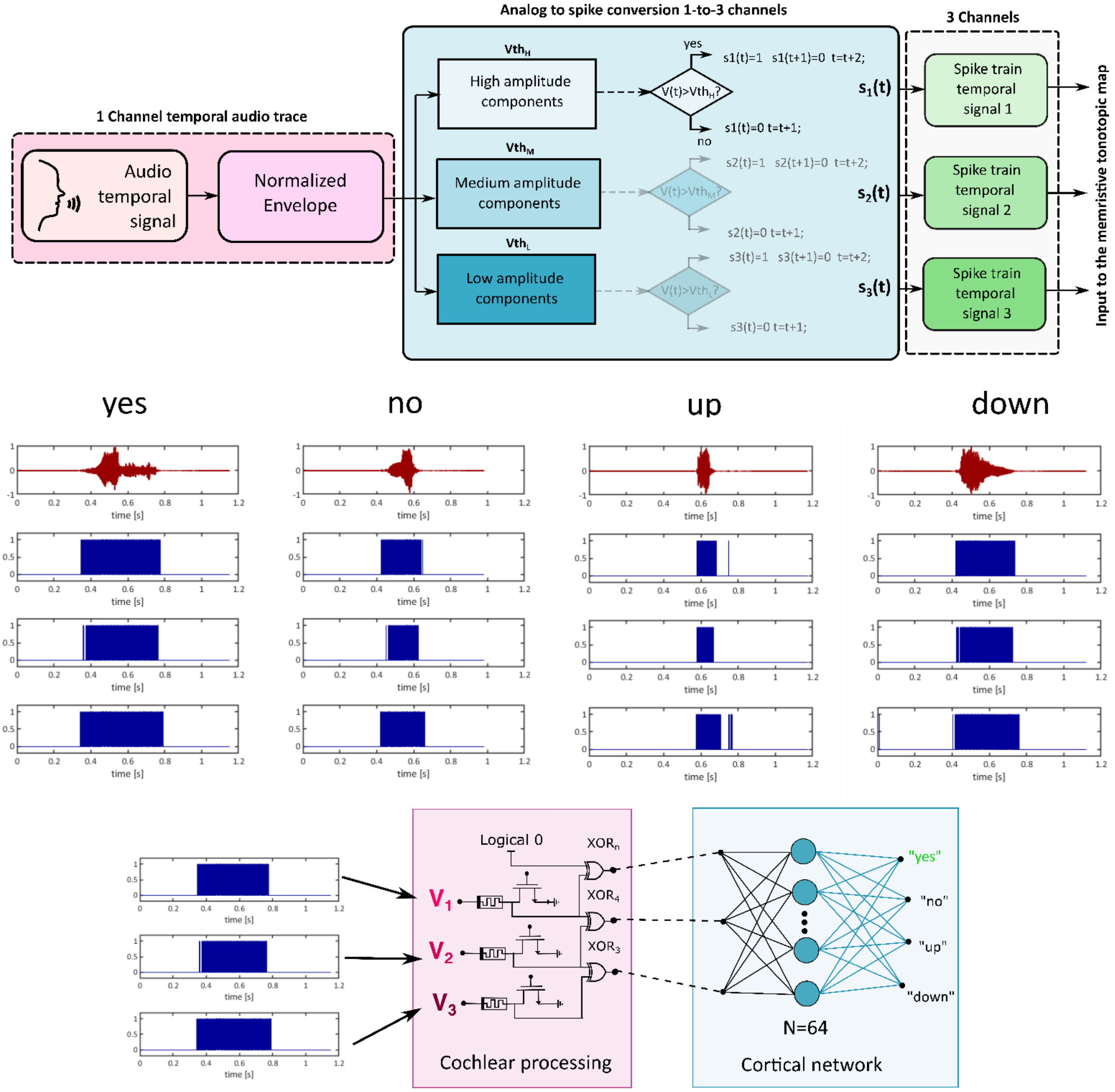

Supplementary Figure 19: Algorithm for Analog-to-spike conversion and resulting trains.

We need to transform the analog temporal audio traces into trains of spike to feed them to MTM. A block diagram of the algorithm is reported in the top part of the Supplementary Figure 19. The audio signal is normalized, and the envelope is extracted. To capture not only different frequency components of different phonemes but also their different amplitude, we used 3 different thresholds for the analog-to-spike conversion. The algorithm checks if the signal at time=t is higher than the threshold. If yes, the associated spike train will have a HIGH value (1) at time=t and LOW value (0) at time=t+1. The zeros are inserted to have equal time duration of the applied spike pulses. If the audio signal is below the threshold, the relative temporal value for spike train will be LOW (0). This process is done for 3 different thresholds. In this way, we obtain that with the higher threshold value just the high amplitude components are detected and transformed into spikes. Lowering the threshold, we also include lower amplitude components in the signal. Results of conversion for different spoken words are reported in the central and bottom part of the Supplementary Figure 19.

Supplementary note 15: CMOS neurons comparison

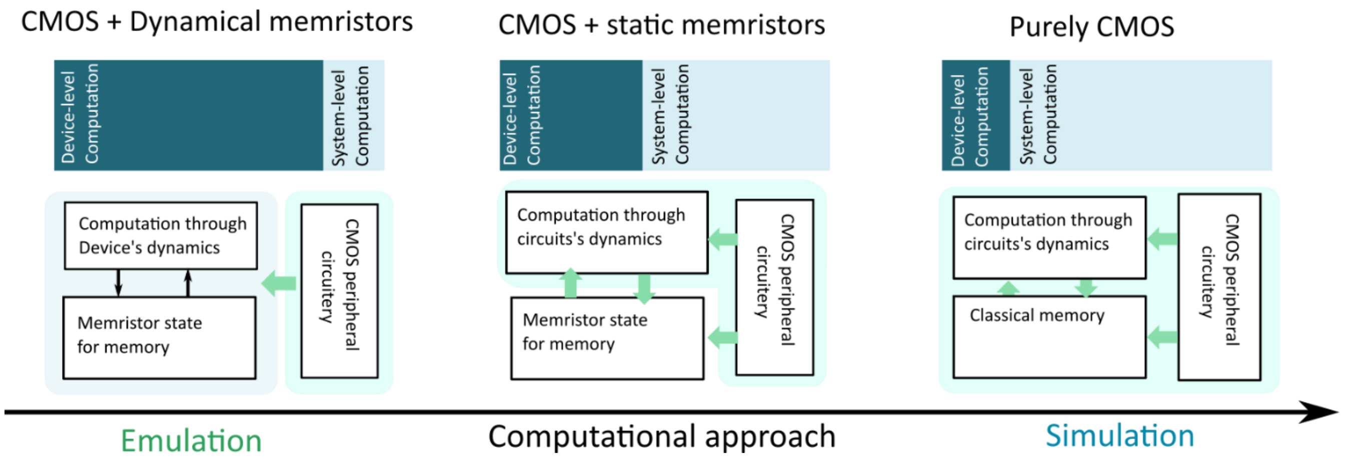

Supplementary Figure 20: Schematic representation of paradigm shift from simulation of biological mechanisms with purely CMOS approach to emulation with dynamical memristors.

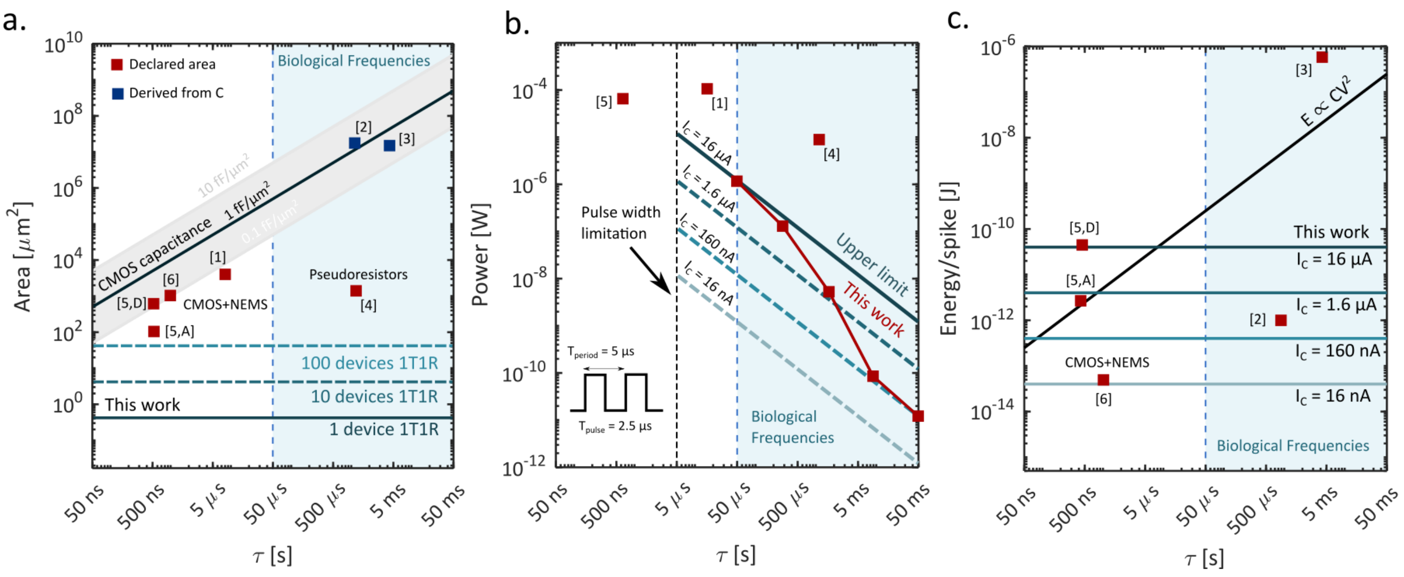

Supplementary Figure 21: Comparison between parameters of a single CMOS leaky integrate-and-fire (LIF) neuron implementation from different works in 65-nm technology and this work. Due to their interesting results, we also included a work that couple CMOS with nano electromechanical systems (NEMS) [6] and a work using pseudoresistors [4] **a.** Area occupation versus characteristic time constant. **b.** Power consumption versus characteristic time constant. c. Energy per spike versus characteristic time constant.

The area occupation of this work is estimated to be the worst case for RRAM technology referring to $100F^2$ [11]. The energy of the single spike is calculated as:

$$E = V_{TE} I_C t_{pulse} \tag{6}$$

The power consumption upper limit is calculated considering that all the pulses in the train cause a switch, being:

$$P_{lim} = \frac{N_{pulses}(\tau)V_{in}I_C t_{pulse}}{T_{window}} = \frac{T_{window}}{\tau}\frac{V_{in}I_C t_{pulse}}{T_{window}} = \frac{E}{\tau} \tag{7}$$

While the real power consumption is calculated by extracting the average number of ON spikes in the experiments for the intermediate voltage amplitude of 1.5V:

$$P = \frac{\overline{N_{pulses}(\tau)}V_{in}I_C t_{pulse}}{T_{window}} \tag{8}$$

Supplementary note 16: 1R I-V curve and 1T1R I-V curve for different current compliances

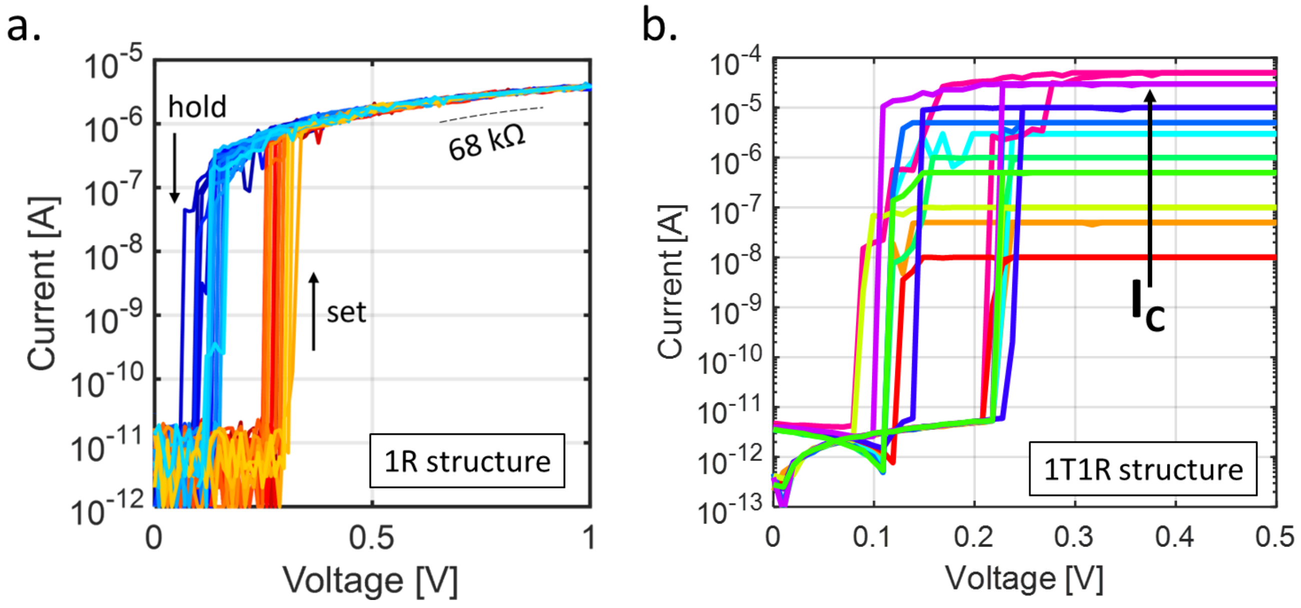

Supplementary Figure 22: Quasi-static I-V curves of volatile RRAM for different structures. a. 1R structure. **b.** 1T1R structure.

References

1. Aamir, Syed Ahmed, et al. "A highly tunable 65-nm CMOS LIF neuron for a large scale neuromorphic system." ESSCIRC Conference 2016: 42nd European Solid-State Circuits Conference. IEEE, 2016.
2. Rozenberg, M. J., O. Schneegans, and P. Stoliar. "An ultra-compact leaky-integrate-and-fire model for building spiking neural networks." Scientific reports 9.1 (2019): 11123.
3. Velichko, Andrei, and Petr Boriskov. "Concept of LIF neuron circuit for rate coding in spike neural networks." IEEE Transactions on Circuits and Systems II: Express Briefs 67.12 (2020): 3477-3481
4. Chen, Xiangyu, et al. "An ultra-compact leaky integrate-and-fire neuron with long and tunable time constant utilizing pseudo resistors for spiking neural networks." Japanese Journal of Applied Physics 61.SC (2022): SC1051.
5. Joubert, Antoine, et al. "Hardware spiking neurons design: Analog or digital?." The 2012 International Joint Conference on Neural Networks (IJCNN). IEEE, 2012.
6. Saha, Sumit, et al. "Energy Efficient Lif Neuron Circuit Using Hybrid Cmos-Nems in 65 Nm Cmos Technology." 2022 IEEE 35th International Conference on Micro Electro Mechanical Systems Conference (MEMS). IEEE, 2022.
7. Calimera A, Macii E, Poncino M. The Human Brain Project and neuromorphic computing. Funct Neurol. 2013 Jul-Sep;28(3):191-6. PMID: 24139655; PMCID: PMC3812737.
8. Indiveri, Giacomo, and Timothy K. Horiuchi. "Frontiers in neuromorphic engineering." Frontiers in neuroscience 5 (2011): 118.
9. Zenke, Friedemann, et al. "Visualizing a joint future of neuroscience and neuromorphic engineering." Neuron 109.4 (2021): 571-575.
10. Pham, Martin Do, et al. "From Brain Models to Robotic Embodied Cognition: How Does Biological Plausibility Inform Neuromorphic Systems?." Brain Sciences 13.9 (2023): 1316.
11. Lepri, N., et al. "In-memory computing for machine learning and deep learning." IEEE Journal of the Electron Devices Society (2023).
12. Peinl, R., Rizk, B., & Szabad, R., “Open source speech recognition on edge devices”, 10th International Conference on Advanced Computer Information Technologies (ACIT), IEEE, (2020).
